# Determinants of Physicians’ Referrals for Suspected Cancer Given a Risk-Prediction Algorithm: Linking Signal Detection and Fuzzy Trace Theory

**DOI:** 10.1177/0272989X251376024

**Published:** 2025-10-16

**Authors:** Olga Kostopoulou, Bence Pálfi, Kavleen Arora, Valerie Reyna

**Affiliations:** Imperial College London, UK; Goldsmiths University of London, UK; Imperial College London, UK; Cornell University, Cornell, NY, USA

**Keywords:** algorithms, decision making, gist, primary care, risk assessment, risk prediction

## Abstract

**Background:**

Previous research suggests that physicians’ inclination to refer patients for suspected cancer is a relatively stable characteristic of their decision making. We aimed to identify its psychological determinants in the presence of a risk-prediction algorithm.

**Methods:**

We presented 200 UK general practitioners with online vignettes describing patients with possible colorectal cancer. Per the vignette, GPs indicated the likelihood of referral (from highly unlikely to highly likely) and level of cancer risk (negligible/low/medium/high), received an algorithmic risk estimate, and could then revise their responses. After completing the vignettes, GPs responded to questions about their values with regard to harms and benefits of cancer referral for different stakeholders, perceived severity of errors, acceptance of false alarms, and attitudes to uncertainty. We tested whether these values and attitudes predicted their earlier referral decisions.

**Results:**

The algorithm significantly reduced both referral likelihood (*b* = −0.06 [−0.10, −0.007], *P* = 0.025) and risk level (*b* = −0.14 [−0.17, −0.11], *P* < 0.001). The strongest predictor of referral was the value GPs attached to patient benefits (*b* = 0.30 [0.23, 0.36], *P* < 0.001), followed by benefits (*b* = 0.18 [0.11, 0.24], *P* < 0.001) and harms (*b* = −0.14 [−0.21, −0.08], *P* < 0.001) to the health system/society. The perceived severity of missing a cancer vis-à-vis overreferring also predicted referral (*b* = 0.004 [0.001, 0.007], *P* = 0.009). The algorithm did not significantly reduce the impact of these variables on referral decisions.

**Conclusions:**

The decision to refer patients who might have cancer can be influenced by how physicians perceive and value the potential benefits and harms of referral primarily for patients and the moral seriousness of missing a cancer vis-à-vis over-referring. These values contribute to an internal threshold for action and are important even when an algorithm informs risk judgments.

**Highlights:**

- Physicians’ inclination to refer patients for suspected cancer is determined by their assessment of cancer risk but also their core values; specifically, their values in relation to the perceived benefits and harms of referrals and the seriousness of missing a cancer compared with overreferring.
- We observed a moral prioritization of referral decision making, in which considerations about benefits to the patient were foremost, considerations about benefits but also harms to the health system or the society were second, while considerations about oneself carried little or no weight.
- Having an algorithm informing assessments of risk influences referral decisions but does not remove or significantly reduce the influence of physicians’ core values.

## Introduction

General practitioners (GPs) are on the front line of referral decisions for suspected cancer. To expedite the earlier detection of cancer, a national priority in the United Kingdom,^1^ GPs can refer patients on the 2-wk-wait (2WW) pathway, so that patients with suspected cancer are seen by a specialist or for specialist investigations within 2 weeks from referral.^2^ In this article, we used the term “referral” to signify 2WW referrals. Signal detection theory suggests that such decisions are determined by 2 factors: “discrimination” and “response bias.”^3,4^“Discrimination” refers to our ability to distinguish between situations that necessitate a specific response or action (e.g., referral for suspected cancer) and those that do not. “Response bias” refers to our inclination to take action and, in theory, is independent of discrimination. Two people may be equally good at discrimination but produce different responses because they differ in their response bias. For example, three social workers may review the same evidence and make the same risk assessment regarding a child in possible need. Yet, one decides to refer the child, the other decides to repeat the assessment, and the third decides to dismiss the case. These three different responses are produced by differences in an internal threshold for acting. The second and third social workers need more evidence (a stronger signal) before deciding to act.

Discrimination and response bias can be measured by recording decisions over multiple trials with known outcomes and estimating the number of correct and incorrect responses. “Hits” are the number of trials in which action should be taken, and action was indeed taken; “false alarms” are the number of trials in which action should not be taken but action was taken. In previous research, we measured the discrimination and response bias of large samples of UK GPs who made referral decisions about hypothetical patients with possible cancer. One study involved clinical vignettes of patients with possible colorectal cancer (*N* = 216),^5^ the other with possible upper gastrointestinal cancer (*N* = 252).^6^ A subset of 165 GPs took part in both studies with an approximately 1-year interval in between studies. Although the average discrimination of these GPs was uncorrelated between studies, there was substantial correlation of average response bias: GPs who were inclined to refer patients for one type of cancer were also inclined to refer patients for the other type of cancer. This suggests that, in addition to—and independent from—risk assessment, some GPs are more inclined than others to refer patients for suspected cancer.

Decision thresholds can determine differences in health care professionals’ decision making more than discrimination or accuracy in risk assessment can.^7,8^ Furthermore, there is evidence that referral thresholds at the level of clinics (GP practices), representing the collective response bias across individual GPs within each clinic, are responsible for missed cancers to a greater extent than discrimination is.^9^ In the present study, we investigate the psychological determinants of individual GPs’ referral decisions, separating risk assessment from bias to take action under uncertainty. Furthermore, given the increasing emphasis on using algorithmic models to reduce clinical uncertainty, we explored whether an algorithm informing GPs of the probability that the patient has cancer influenced both risk assessment and referral responses.

In theory, GPs could base their decisions on Swets et al.’s “optimal threshold” equation.^10^ The optimal threshold (i.e., the one that maximizes benefits relative to costs) depends on the base rates of signal and nonsignal events (e.g., cancer present v. absent) and how the decision maker evaluates the expected outcomes of action and inaction, that is, the benefits of a correct response (hits and correct rejections) and the costs of an incorrect response (misses and false alarms). However, it is unlikely that GPs perform a deliberative cost-benefit analysis when deciding whether to refer a patient for suspected cancer.

Instead, psychological research indicates that perceived harms and benefits exert their influence via an intuitive mode of thinking (for a review, see Reyna and colleagues^11^). According to fuzzy trace theory, decision makers encode mental representations of the gist of information (e.g., “this risk is high!”) in parallel with verbatim details (e.g., “the risk is 15%”). Gist captures the bottom-line meaning, beyond specific words, pictures, or numbers.^12^ The predominant gist can be identified with well-structured questionnaires, and it has been found to predict health care decision making (e.g., antibiotics prescribing).^13^ Across studies, the gist of risk often boils down psychologically to fuzzy ordinal distinctions, such as none/negligible, low, moderate, and high. To reach a decision, similarly gist-based values, a kind of personal aphorisms, are applied to the mental representations, such as “better safe than sorry” or “it is bad to refer low-risk patients” or “missing a cancer is the worst possible thing.” Therefore, in this study, we elicited GPs’ assessments of risk using not numerical but gist-type response scales with categories ranging from negligible to high. We also elicited their values and attitudes with regard to a host of variables that we expected to predict referral responses.

## Methods

### Approach, Aim, and Hypotheses

We ran the study online and recruited GPs to respond to clinical vignettes, which had been used in a previous study that demonstrated the impact of algorithmic risk advice on GPs’ numerical risk assessments and referral responses.^14^ Per vignette, GPs made categorical judgments of risk and referral likelihood both before and after seeing the output of an algorithm. Based on our previous study, we expected that both referral and risk responses would change significantly following algorithmic advice (*H*1) and that referral responses would become more appropriate with regard to the 3% National Institute for Health and Care Excellence (NICE) referral threshold^i^ (*H*2).^15^ After all the vignettes were completed, we elicited 1) perceptions of global harms and benefits of referral for patients, the health system/society, and GPs themselves/their clinic at different levels of cancer risk; 2) how GPs viewed missing a cancer compared to overreferring; 3) their acceptance of overreferring for detecting a single cancer; and 4) their attitudes toward clinical uncertainty. We aimed to explore how these global values and attitudes predicted GPs’ earlier responses on the clinical vignettes. Specifically, we expected the following associations:

1. positive associations between the likelihood of referring the patients in the vignettes and
  - perceived global benefits of referrals (*H*3),
  - perceived severity of missing a cancer vis-à-vis overreferring (*H*4), and
  - willingness to trade false-positive referrals for one cancer detection (*H*5); and
2. negative associations between the likelihood of referring the patients in the vignettes and
  - perceived global harms of referrals (*H*6), and
  - tolerance of clinical uncertainty (*H*7).

### Sample Size Estimation and Participant Recruitment

We powered the study for a multilevel linear regression to test whether perceived global harms and benefits of referrals can predict referral of the vignettes. Using G*Power 3.1.9.7, we estimated that a minimum of 652 independent responses would be required to detect a small effect (*f*^2^ = 0.02) using 2-tailed tests with alpha of 0.05 and 95% power in a multiple linear regression. We adjusted this number by the design effect (DE) for data clustering.^16^ The DE was calculated using the formula DE = 1 + (*n*− 1) × ICC, where *n* is the cluster size, that is, the number of vignettes to be completed by each GP (9 vignettes), and ICC is the intraclass correlation, which was estimated to be 0.116 based on the previous study that used the same dependent variable.^14^ Hence, DE equals 1.928. To calculate the minimum number of GPs we would need, we multiplied the required number of independent responses by the DE and divided it by the cluster size (652 × 1.928/9 = 140). Therefore, we estimated that we would need to recruit at least 140 GPs.

Participation in this study was limited to fully qualified GPs and GP trainees at their final stage of specialty training (i.e., ST3 and above) currently practicing in England. We recruited from our database of GPs who had participated in previous decision-making studies by our research group and had indicated an interest in taking part in similar, future studies. We invited 596 eligible GPs who had not participated in our previous study on risk algorithms in colorectal cancer referral.^14^ The invitation e-mail briefly introduced the study and the benefits of participation (Amazon voucher of £30) and included a link to an expression-of-interest form, where GPs could sign up for the study by providing their National Health Service (NHS) e-mail address. A total of 255 GPs completed this expression-of-interest form (43% response rate). Data collection was undertaken between February 12, 2022, and October 4, 2023 (dates of first and last completion).

### Materials

We slightly adapted 12 clinical vignettes that we had used in the previous study of referral decision making.^14^ Each vignette described a hypothetical patient presenting to the GP with a combination of risk factors and symptoms that could indicate colorectal cancer. Three of the vignettes were used for practice purposes. These vignettes had risk scores of 1.04% (low), 6.33% (moderate), and 39.58% (high), as calculated using a publicly available cancer risk calculator (https://www.qcancer.org). The remaining 9 vignettes were used for data collection. Three of these were of low risk (1% to 2%), 3 were of moderate risk (5% to 9%), and 3 were of high risk (21% to 40%).

All vignettes started with the patient demographics and risk factors presented in a list format: patient name and gender, age, body mass index, smoking status (never smoked/ex-smoker/number of cigarettes per day), alcohol intake (units/week), and age of menopause for female patients younger than 60 y. Each vignette included symptoms and some nonclinical, filler information in a narrative format. The latter was intended to make the vignettes more realistic and engaging for the participants. Each vignette finished with a statement that there were no other symptoms and that examination findings were normal. The vignettes with their estimated risk and appropriate referral responses are presented in Supplement 1.

### Design and Procedure

The study followed a pre-post design, with response timing (pre- v. post-algorithm) as the within-participant factor. We used the Qualtrics XM platform (Qualtrics, Provo, UT) to create an online survey that the GPs could access via a link that they received after they signed up to the study. Initially, GPs read detailed information about the study and provided online consent. They then completed basic demographic information (gender, age, GP status [fully qualified or trainee], and year of qualification). Before seeing the vignettes, GPs were presented with information about the algorithm’s derivation, validation, and accuracy (see Kostopoulou et al., p. 3, box 1, for the exact wording^14^). Next, they were provided with the 3 practice vignettes in a random order to familiarize them with the interface and task. The procedure was identical for all vignettes.

After the practice session was over, GPs were presented with the 9 remaining vignettes in a random order. At the end of each vignette, they were asked to respond to 2 questions in the following order:

*How likely is it that you would refer this patient for specialist investigations within 2 weeks and/or refer on the 2WW suspected colorectal cancer pathway at this consultation?* (highly unlikely, unlikely, uncertain, likely, highly likely)

*In your clinical judgment, which of the following best describes the risk of colorectal cancer for this patient?* (negligible, low, medium, high)

Although we would expect risk assessment to precede a referral decision, we chose to ask about risk only after clinicians responded to the referral question. In this way, we felt that we would obtain more gistlike referral responses, not diluted by explicit elicitations of risk.

**Figure 1:**
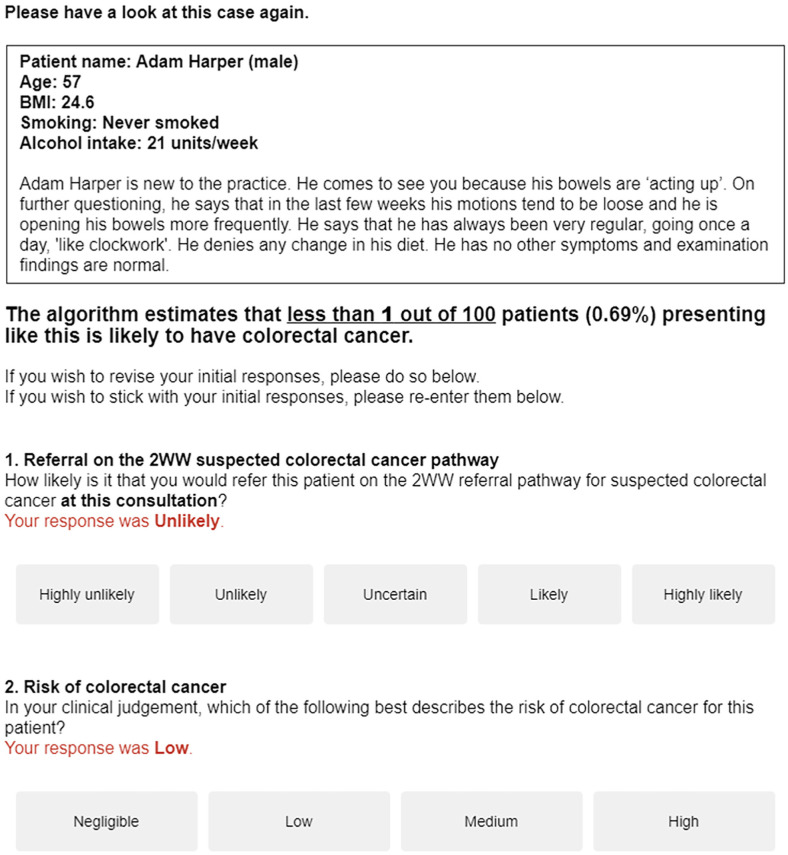
Screenshot of a vignette presented again to a respondent, this time with the algorithmic risk estimate and a reminder of the initial responses.

After responding to these 2 questions, the same vignette was presented again with the corresponding algorithmic score in frequency and percentage formats. GPs were reminded of their own responses and were invited to revise them, if they wished, or reenter them (Figure 1).

After all 9 vignettes were completed, we asked GPs a series of general questions not referring specifically to the vignettes seen earlier and in the following sequence:

#### Step 1

**Figure 2:**
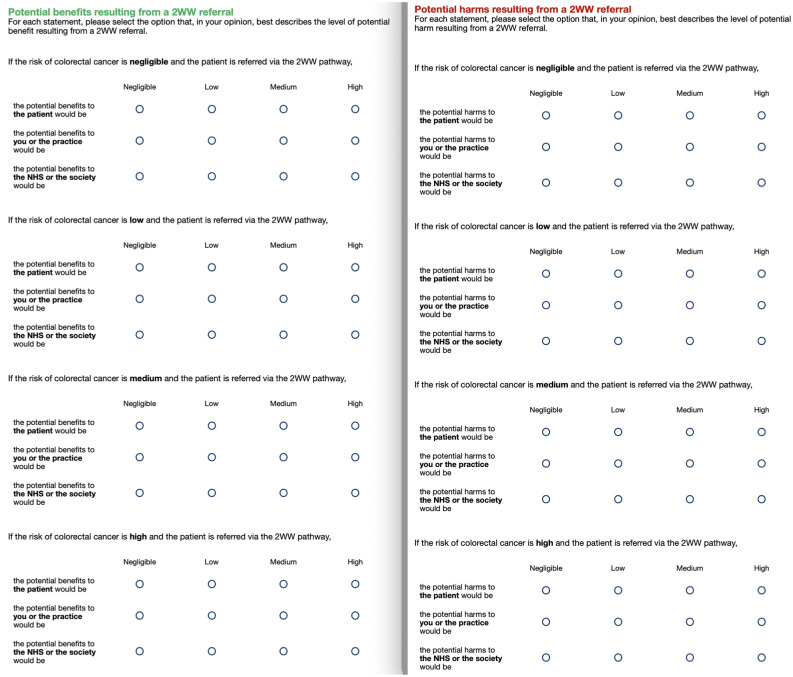
Rating scales for perceived harms (left-hand side) and benefits (right-hand side) for 4 gist levels of cancer risk and 3 stakeholders. The harm questions always preceded the benefits questions.

First, we asked GPs to rate potential global harms and benefits of 2WW referrals at each of 4 gist levels of cancer risk corresponding to the scale used to assess risk in the vignettes: negligible, low, medium, and high. For each risk level, we asked them to consider 3 stakeholders: the patient, the NHS or the society, and the GP or the practice (representing patient-centered, public-centered, and self-centered values, respectively). Thus, we elicited 12 ratings for potential benefits and 12 ratings for potential harms. Ratings were given on 4-point gist scales (negligible, low, medium, high; see Figure 2).

#### Step 2

**Figure 3:**
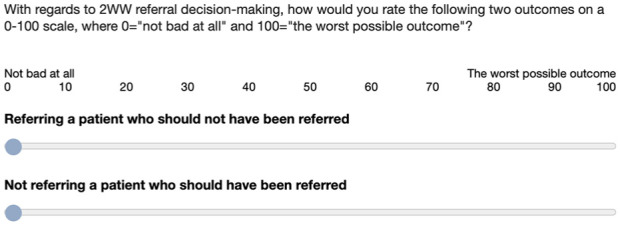
Rating scales for measuring the perceived severity of false alarms and misses.

Next, we measured the perceived severity of 2 types of errors (i.e., how bad GPs thought they were) in relation to 2WW referrals: false alarms (referring a patient who should not have been referred) and misses (not referring a patient who should have been referred). Responses were given on separate 0 to 100 scales (Figure 3). Note that we asked about “outcomes” rather than “errors” to avoid the negative loading of the term “error” and because referring a patient who should not be referred may not be considered an error. Reyna and Lloyd^17^ used a similar question about admission to the hospital of patients with unstable angina but asked physicians to make a direct comparison between false alarms and misses (“which of the two errors is worse?”) on a 0 to 100 scale ranging from *no difference at all* to *the maximum possible difference*.

#### Step 3

**Figure 4:**
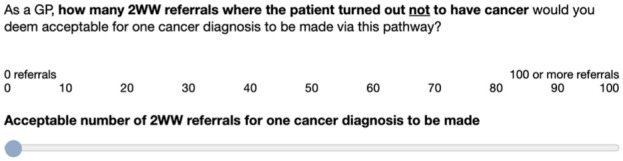
Rating scale for measuring willingness to trade false alarms for a hit.

Then, we measured the willingness to trade false alarms for a hit by asking GPs to state how many 2WW referrals, in which the patient turned out not to have cancer, they would deem acceptable for one cancer diagnosis to be made via this pathway (Figure 4).

#### Step 4

Finally, we measured the attitudes toward risk and uncertainty in clinical practice using a validated questionnaire consisting of five items measured on 5-point Likert-type scales ranging from 1 (*strongly disagree*) to 5 (*strongly agree*).^18^ Items concerned not taking any risks with physical complaints, seeking certainty about patients’ diagnoses, referring to a specialist rather than wait and see, doing everything one can to establish the cause of a complaint, and awareness that the complaint can be the beginning of a serious disease. At the end, participants had the opportunity to provide written feedback on any aspect of the study.

### Statistical Analyses

#### Creation of variables

Referral responses on the 9 vignettes were scored from −2 (*highly unlikely*) to +2 (*highly likely*). Judgments of risk were scored from 0 (*negligible*) to 3 (*high*). To test *H*1, we created 2 binary variables, one for referral responses and the other for risk judgments, which indicated whether GPs changed their responses post algorithm (0 to indicate no change and 1 to indicate change).

To measure the appropriateness of referral responses (*H*2), we used the NICE risk threshold of 3%: in vignettes with a QCancer risk of more than 3%, we categorized *likely* and *highly likely* referral decisions as appropriate and *unlikely* and *highly unlikely* ones as inappropriate; in vignettes with a QCancer risk of less than 3%, we categorized *unlikely* and *highly unlikely* referral decisions as appropriate and *likely* and *highly likely* ones as inappropriate.

To measure the perceived error severity, we subtracted the ratings of false alarms from those of potential misses. Scores could vary from +100 to −100, however, we expected misses to be considered more severe than false alarms and hence most scores to be positive. For the willingness-to-trade question, we used the raw 0 to 100 values, indicating the number of false alarms GPs would accept for one cancer to be detected. To create a single attitude-to-uncertainty score for each participant, we averaged responses across the five questions of the Grol et al.^18^ questionnaire and reversed the final score so that high values denoted willingness to tolerate uncertainty.

#### Regression analyses

All regression analyses were multilevel with random intercepts by GP and either vignette or risk level, depending on the model. To test the regression slopes, we used significance testing with the traditional *P* value threshold of 0.05 and reported the regression coefficients and 95% confidence intervals [CIs]. The reported regression models of referral responses and risk judgments are all linear, but because the response variable is ordinal, we also ran ordinal models to assess the robustness of our findings. Notably, the results of the ordinal models are in harmony with those reported here (see Supplement 5). We conducted the analyses in R (version 4.3.1) and confirmed them in STATA 17.0. For the nonsignificant statistical tests, we used the Bayes factor (BF)^19,20^ to distinguish between data insensitivity and evidence for the alternative versus null hypotheses (see Supplement 2 for more details).

## Results

### Study Sample

We recruited 200 GPs (193 fully qualified and 7 trainees). Their mean age was 42 y (*s =* 8.5), and 52% were female (103/200). The sample’s average experience was 12 y in general practice post-qualification (*s =* 8.6, median = 9, range = 0–39). Five GPs reported 1- or 2-digit numbers rather than their year of qualification, so they were not included in the count or in any analyses involving the experience variable.

### Algorithm Impact on Risk and Referral Responses

Both referral and risk responses changed significantly post-algorithm (referral change v. no change odds ratio [OR] 0.25 [0.16, 0.40], *P* < 0.001; risk change v. no change OR 0.49 [0.35, 0.59], *P* < 0.001; see Table 1), thus confirming *H*1. When we regressed the referral responses on timing (pre- v. post-algorithm), we found that the likelihood of referral reduced post-algorithm (*b* = −0.06 [−0.10, −0.007], *P* = 0.025). Similarly, when we regressed risk judgments on timing, we found that they too significantly reduced post-algorithm (*b* = −0.14 [−0.17, −0.11], *P* < 0.001).

**Table 1:** Frequencies and Within-Column Relative Frequencies (%) of Referral and Risk Response Categories Pre- and Post-algorithm

Outcome Variable	Response	Pre-algorithm	Post-algorithm	Total
Referral	Highly unlikely (−2)	63 (3.5%)	88 (4.9%)	151 (4.2%)
	Unlikely (−1)	278 (15.4%)	320 (17.8%)	598 (16.6%)
	Uncertain (0)	291 (16.2%)	236 (13.1%)	527 (14.6%)
	Likely (1)	431 (23.9%)	416 (23.1%)	847 (23.5%)
	Highly likely (2)	737 (40.9%)	740 (41.1%)	1,477 (41.0%)
Risk	Negligible (0)	35 (1.94%)	79 (4.39%)	114 (3.2%)
	Low (1)	479 (26.61%)	575 (31.94%)	1,054 (29.3%)
	Medium (2)	667 (37.06%)	596 (33.11%)	1,263 (35.1%)
	High (3)	619 (34.39%)	550 (30.56%)	1,169 (32.5%)
	Total	1,800	1,800	3,600 (100.0%)

Examining response changes in more detail, we found that referral responses remained unchanged 75% of the time (1,356/1,800), but when they changed, they moved more often toward no referral (268 times) than toward referral (176 times). Similarly, risk judgments remained unchanged 65% of the time (1,176/1,800), but when they changed, they moved more toward lower risk (429 times) than higher risk (195 times). Although GPs were more conservative with their referrals and did not change them as frequently as risk judgments, risk and referral responses were tightly linked, such that when risk level increased by a unit, referral was more likely by almost a unit: *b* = 0.80 [0.76, 0.84], *P* < 0.001.

These results are comparable with those by Kostopoulou et al.,^14^ in which GPs saw 20 vignettes depicting patients with possible colorectal cancer and responded on the same 5-point referral scale after they had provided a numeric risk estimate on a 0 to 100 visual analog scale (VAS). This suggests that differences in the risk response scales (5-point response scale in this study v. 0–100 VAS in the previous study) and in the design (referral responses requested before v. after risk assessment) did not alter the direction of the results.

### Algorithm Impact on Referral Appropriateness

When we excluded “uncertain” responses from the analysis, we found that 84.6% (1,205/1,425) of responses pre-algorithm and 86.5% (1,232/1,425) post-algorithm were appropriate. This increase was not significant in a multilevel logistic regression (OR 1.28 [0.99, 1.65], *P =* 0.058). However, the Bayesian analysis revealed good enough evidence for the alternative hypothesis, *H*2 (BF_H(0, 1.26)_ = 3.62, RR_BF**>**3_ [1.13, 1.49]). When we included “uncertain” responses as inappropriate in the model, we detected a significant improvement from 68.7% (1,236/1,800) pre-algorithm to 74.7% (1,345/1,800, OR 1.52 [1.28, 1.81], *P* **<** 0.001) post-algorithm. That is, the odds of a NICE-concordant referral response increased by about 50% post-algorithm, mainly due to reduced uncertainty and movement toward not referring.

### Perceived Harms and Benefits as a Function of Risk Level

**Figure 5:**
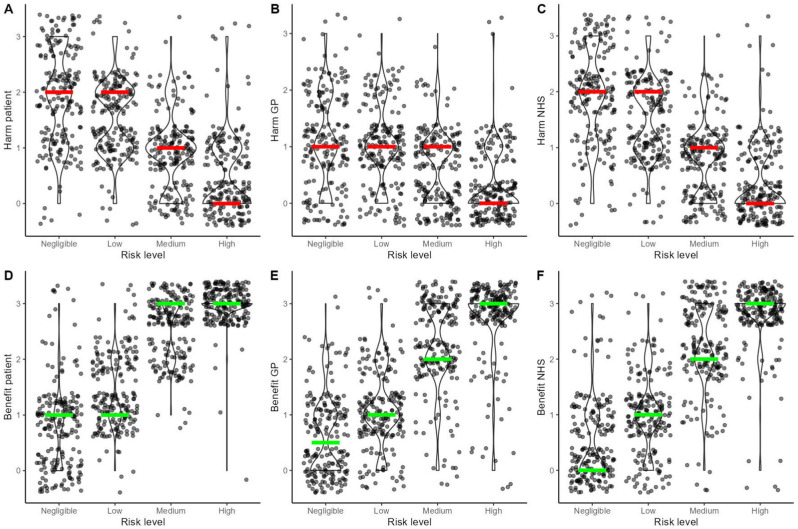
Violin and scatterplots depicting the relationship between the stipulated level of colorectal cancer risk and the perceived potential harms and benefits of 2-wk-wait (2WW) referrals to the patient (A and D), the general practitioner or the practice (B and E), and the National Health Service or the society (C and F). The colored lines highlight the medians per level of risk (red lines for harms and green lines for benefits).

To examine whether GPs’ perception of potential harms and benefits of referrals were sensitive to the category of stipulated cancer risk (i.e., the risk level of each vignette by design), we ran 6 multilevel linear regression models with random intercept by GP, where we regressed the harms and the benefits for each stakeholder (patient, GP/practice, NHS/society) separately on the stipulated risk level. As expected, perceived harms reduced as cancer risk increased (harms to patients: *b* = −0.49 [−0.52, −0.45], *P* < 0.001; harms to GP/practice: *b* = −0.28 [−0.31, −0.25], *P* < 0.001; harms to NHS/society: *b* = −0.53 [−0.57, −0.50], *P* < 0.001), while perceived benefits increased as cancer risk increased (benefits to patients: *b* = 0.76 [0.73, 0.80], *P* < 0.001; benefits to GP/practice: *b* = 0.75 [0.71, 0.78] *P* < 0.001; benefits to NHS/society: *b* = 0.80 [0.77, 0.85], *P* < 0.001) (Figure 5).

### Perceived Harms and Benefits as Predictors of Referral Responses

Next, we tested whether perceived global harms and benefits predicted referral responses on the vignettes, our main research question. Since the harms and benefits questions were general and not about a specific vignette, we linked the harm and benefit ratings to referral responses via the GPs’ judged risk for each vignette. For example, if a GP indicated that the risk of colorectal cancer for a vignette was medium, then we matched their referral response for that vignette with their ratings of global harms and benefits for the medium level of risk. In a multilevel regression model with random intercepts for GP and vignette, we regressed all referral responses on the perceived benefits and harms for each stakeholder and included response timing (pre- v. post-algorithm) as a factor (Table 2). We assessed multicollinearity using the mean variance inflation factor (VIF). This was 3.48 (i.e., lower than 5), and no individual VIF was larger than 5, which suggested that collinearity was not a problem. Perceived global benefits and harms for the patient and the NHS or the society, respectively, predicted referral likelihood in the expected direction, while we found no evidence for perceived global benefits (BF_H(0, 0.15)_ = 0.52, RR_3>BF>1/3_ [0, 0.24]) and global harms for the GP/practice (BF_H(0, 0.15)_ = 0.52, RR_3>BF>1/3_ [0, 0.28]). Thus, *H*3 and *H*6 were supported for the patient and the health system/society but not for the GP. Perceived global patient benefits were the strongest predictor of referral likelihood. When we included in the model the judged level of risk associated with each referral response (i.e., the risk level that the GP assigned to a vignette after providing a referral response), global patient benefits, NHS benefits, and NHS harms remained significant, although their predictive power weakened: *b* = 0.15 [0.09, 0.21], *P* < 0.001 for patient benefits; *b* = 0.09 [0.02, 0.15], *P* = 0.007 for NHS benefits; and *b* = −0.09 [−0.15, −0.02], *P* = 0.007 for NHS harms. Patient harms did not pass the conventional threshold of the 0.05 level of significance in that model (BF_H(0, 0.15)_ = 0.36, RR_3>BF>1/3_ [0, 0.16]). When we included interactions with timing in the model, we found no evidence that the algorithm moderated the impact of perceived global benefits and harms on vignette referrals, even though regressing separately pre-algorithm referrals and post-algorithm referrals on global benefits and harms showed small drops in the regression coefficients post-algorithm (see Supplement 3).

**Table 2:** Regression Coefficients, 95% Confidence Intervals, and *P* Values of a Multiple Regression Model Predicting Referral Responses

Stakeholder	Predictor	Coefficient	95% CI	*P*
Patient	Benefits	0.30	0.23, 0.36	<0.001
	Harms	−0.09	−0.15, −0.02	0.008
NHS/society	Benefits	0.18	0.11, 0.24	<0.001
	Harms	−0.14	−0.21, −0.08	<0.001
General practitioner/practice	Benefits	0.03	−0.03, 0.09	0.36
	Harms	−0.03	−0.10, 0.04	0.41
	Timing (post-algorithm)	0.02	−0.02, 0.07	0.30

Some GPs left written comments at the end of the survey. Some comments in relation to the harm/benefit questions indicated differences in how GPs conceptualized these and some difficulties thinking about referrals in those terms (see Supplement 4). One GP did not provide any ratings of harms for any of the three stakeholders. There were also missing values for certain levels of risk for GP/practice harms (two GPs), NHS/society harms (one GP), and GP/practice benefits (one GP), suggesting that some respondents had difficulty thinking about referral benefits for GPs and referral harms in general. There were no missing values for patient benefits and harms nor for NHS/society benefits.

### Perceived Error Severity, Tradeoffs, and Attitudes to Uncertainty as Predictors of Referral Responses

**Figure 6:**
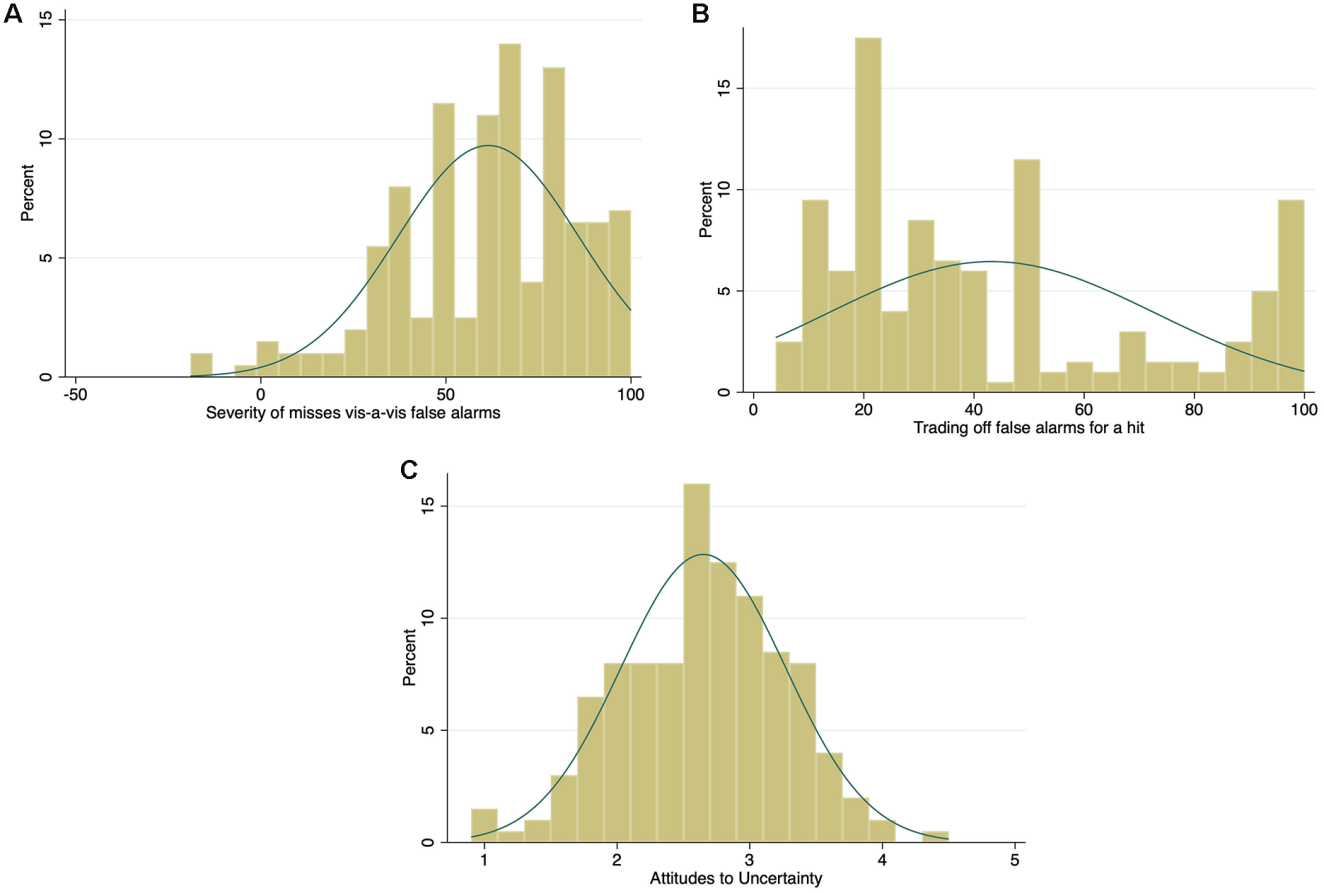
Histograms of the distribution for the variables of perceived error severity (A), tradeoffs (B) and attitudes to uncertainty (C) with normal-density plot lines.

The difference in the perceived severity between potential misses and false alarms (measured at step 2) followed a highly negatively skewed distribution (*median* = 65, *mean* = 61.5, *s =* 24.4, range = −19 to 100; Figure 6A), suggesting that, as expected, respondents perceived the possibility of missing a cancer as more serious than overreferring. Three respondents produced negative values (overreferring more serious than misses), and three others produced 0 values (no difference), which could be the result of misinterpretation or inattentiveness. No responses were dropped from the analyses, however.

The tradeoff variable (measured at step 3) was positively skewed (*median* = 33, *mean* = 43.2, *s =* 29.7) but with a small peak at very high values (Figure 6B). That is, on average, GPs would be willing to refer 43 patients who turned out not to have cancer in order to detect one cancer, but a substantial minority (17%, 34/200) indicated that they would accept 90 or more referrals for a single cancer diagnosis. This pattern of a peak close to the maximum of the response scale, with additional peaks at 50 and 20, suggests underlying categorical or ordinal gist representations rather than smooth continuous quantities.

Attitudes to clinical uncertainty and risk (measured at step 4) followed a normal distribution, with GPs being slightly risk avoiding on average (*mean* = 2.7, *s =* 0.6 on the 1–5 scale; Figure 6C).

Difference in the perceived severity of errors (misses v. false alarms) positively correlated with tradeoffs (*r* = 0.26, *P* < 0.001). There was also a significant correlation between tradeoffs and uncertainty attitudes (*r* = 0.092, *P* < 0.001). When we included each of these related measures in separate regressions, only the difference in perceived error severity was a significant predictor (*b* = 0.004 [0.001, 0.007], *P* = 0.009), such that the greater seriousness GPs attached to missing a cancer compared with overreferring, the more likely they were to refer. Thus, we found support for *H*4.^ii^

When we added perceived error severity to the large model shown on Table 2 and included the judged level of risk (risk gist), perceived error severity remained a statistically significant predictor of referrals (Table 3). Results remained unchanged, with no further significant relationships detected, when GP gender and years of experience were also added to the model.

**Table 3:** Regression Coefficients, 95% Confidence Intervals, and *P* Values of a Multiple Regression Model Predicting Referral Responses

Stakeholder	Predictor	Coefficient	95% CI	*P*
Patient	Benefits	0.15	0.09, 0.21	<0.001
	Harms	−0.02	−0.08, 0.04	0.58
National Health Service/society	Benefits	0.08	0.02, 0.15	0.008
	Harms	−0.09	−0.15, −0.02	0.008
General practitioner/practice	Benefits	−0.04	−0.10, 0.02	0.21
	Harms	−0.02	−0.08, 0.05	0.66
	Severity of misses vis-à-vis false alarms	0.003	0.0009, 0.006	0.007
	Risk gist	0.56	0.50, 0.63	<0.001
	Timing (post-algorithm)	0.05	0.01, 0.10	0.011

## Discussion

Our study sheds light on psychological factors that make clinicians (dis)inclined to refer patients with suspected cancer, namely, their intuitive judgments of risk and their core values about potential errors of referring or not referring patients. Perceived benefits and harms to the patient and the health system or society all predicted the likelihood of referring, with benefits for the patient exerting the strongest influence. In contrast, benefits and harms to the clinician or their clinic did not appear to affect the likelihood of referral, suggesting that GPs’ values were patient centered rather than self centered. Although GPs may be subject to pressures from colleagues or the health system to refer more or fewer patients, they did not appear to give such considerations much weight relative to the well-being of the patient. Our findings are consistent with a moral prioritization of referral decision making, in which perceived benefits to the patient have the strongest impact, followed by perceived benefits and harms to the health system and society, followed by little to no weight given to self. The size of the regression coefficients clearly illustrates this moral pyramid of duty: the doctor’s duty to save lives and not to consider reputational or cost implications to themselves and their practice.

In health care systems with salient resource constraints (i.e., in which the care of individual patients can directly or indirectly affect the resources available to other patients), physicians may take both an individual-patient and a population/public-health perspective and trade these off against one another.^21^ A moral dilemma occurs when physicians face a conflict between maximizing collective (or personal) interests against the interests of their individual patients. Although physicians are no doubt aware of resource constraints, in the current study, the perceived benefits to the individual patient primarily guided referral decisions. Thus, decision making in this context appears more consistent with a deontological (core values) perspective, which eschews moral tradeoffs, rather than a utilitarian perspective, which focuses on maximizing the greatest good for the greatest number.

When dealing with patients in everyday clinical practice, benefit and harm considerations could play a different role from those we observed, and considerations of oneself and the practice may indeed influence referrals. Anecdotally, UK GPs acknowledge conflicting pressures from health authorities to refer more patients and communications from hospitals suggesting that they refer inappropriately (i.e., false alarms). In addition, metrics of a practice’s 2WW referral performance used to be in the public domain, so that practices could compare themselves with the best performers, receiving a kind of norm nudging.^22–24^ It is possible that such pressures may influence the response bias of individual GPs, but our study did not detect this, as other variables exerted a larger influence. Hypothetical judgments might also have precluded or minimized consideration of external pressures. Had we made explicit those pressures before GPs responded to the vignettes (e.g., “your practice’s referral rate is below average”), we might have observed an impact not only on referral responses but also on self-interest as a predictor of those responses.

Even when we included in the model the judged level of risk, a variable closely and directly associated with referral responses, physicians’ values regarding patient benefits and NHS/societal benefits and harms continued to predict referral responses. The algorithm consistently lowered the perceived risk and referral likelihood without overriding the impact of perceived benefits and harms. A trend to attenuate them was nevertheless observed, which could reflect the algorithm’s pressure to unmoor physicians from their perceptions and core values. Had the algorithm also provided an explicit recommendation, this could have increased the pressure on physicians to conform to algorithmic advice despite possible misgivings.

We also found that the more physicians considered missing a cancer as a more serious error than referring a patient who turns out not to have cancer, the more likely they were to refer. This relationship was, however, not confirmed by the number of false alarms they were willing to accept for a single cancer diagnosis. This attests to the difference the simple wording of questions can make. Fuzzy trace theory suggests that these exact numerical responses are incompatible with preferred modes of thinking even in numerate populations such as physicians.^11^

Decision thresholds have also been linked to emotions such as anticipated regret and anticipatory worry.^25^ A future study could attempt to measure these emotions directly and assess their effect on referral decisions.

Beyond mandates and incentives, which are known to influence response bias, sometimes coercively, attempts to influence decision making could also target decision makers’ cost/benefit perceptions.^26^ We did not try to identify precisely which harms and benefits respondents had in mind when answering the questions, and it is likely that these differed among respondents, as suggested by some comments. For example, one clinician may value the peace of mind that patients may gain from a referral that is unlikely to find a cancer, while another may wish to save these patients from the pain and stress of unnecessary investigations (but see Nurek and Kostopoulou^27^ about the value patients place on invasive tests that can help to exclude a rare but serious disease). Similarly, one GP may consider that a high referral rate could save the health system money in the long run by identifying and treating cancers early, while another may focus on the potential harm to other patients from increasing waiting lists. We could, of course, have given our respondents examples of harms and benefits but did not want to preempt what they considered important or suggest to them issues that they had not considered before. Our findings could be used to validate studies based on self-reports of harms and benefits, such as interviews. For example, we would expect that all GPs would mention referral benefits to patients, while very few, if any, would voice considerations about themselves or their practice.

## Conclusion

Algorithmic risk estimates appear to influence physicians’ perceived risks and referrals for cancer patients. However, they do not supplant core values about the benefits and harms from a referral and the moral seriousness of missing a cancer vis-à-vis overreferring. These values are thought to contribute to an internal threshold for action that prioritizes benefits to the patient while only secondarily considering benefits and harms to the health system or society and negligibly considering the self.

## Supplemental Material

sj-pdf-1-mdm-10.1177_0272989X251376024 – Supplemental material for Determinants of Physicians’ Referrals for Suspected Cancer Given a Risk-Prediction Algorithm: Linking Signal Detection and Fuzzy Trace Theory Supplemental material, sj-pdf-1-mdm-10.1177_0272989X251376024 for Determinants of Physicians’ Referrals for Suspected Cancer Given a Risk-Prediction Algorithm: Linking Signal Detection and Fuzzy Trace Theory by Olga Kostopoulou, Bence Pálfi, Kavleen Arora and Valerie Reyna in Medical Decision Making

## Notes

i. The NICE referral threshold of 3% (0.03) is the positive predictive value, that is, the probability that a patient with a specific symptom or presentation has cancer. This means that, on average, (1/0.03=) 33 people need to be referred for 1 cancer to be diagnosed.

ii. The Bayesian analysis showed data insensitivity for the tests of tradeoffs (BF_H(0, 0.004)_ = 0.56, RR_3>BF>1/3_ [0, 0.007]) (*H*5) and attitudes to uncertainty (BF_H(0, 0.10)_ = 0.79, RR_3>BF>1/3_ [0, 0.11]) (*H*7).

## References

[1] Crosby D Lyons N Greenwood E , et al. A roadmap for the early detection and diagnosis of cancer. Lancet Oncol. 2020;21(11):1397–9. DOI: 10.1016/S1470-2045(20)30593-3 PMC7535618 33031732

[2] Round T Gildea C Ashworth M. Association between use of urgent suspected cancer referral and mortality and stage at diagnosis: a 5-year national cohort study. Br J Gen Pr. 2020;70(695):e389–98. DOI: 10.3399/bjgp20X709433 PMC7176359 32312762

[3] Macmillan N Creelman C. Detection Theory: A User’s Guide. Mahwah (NJ): Lawrence Erlbaum Associates; 2005.

[4] Stanislaw H Todorov N. Calculation of signal detection theory measures. Behav Res Methods Instrum Comput. 1999;31(1):137–49. 10.3758/bf03207704 10495845

[5] Kostopoulou O Nurek M Cantarella S Okoli G Fiorentino F Delaney BC. Referral decision making of general practitioners: a signal detection study. Med Decis Making. 2019;39(1):21–31. DOI: 10.1177/0272989X18813357 30799690 PMC6311616

[6] Kostopoulou O Nurek M Delaney BC. Disentangling the relationship between physician and organizational performance: a signal detection approach. Med Decis Making. 2020;40(6):746–55. DOI: 10.1177/0272989X20936212 PMC7457451 32608327

[7] Cheyne H Dalgleish L Tucker J , et al. Risk assessment and decision making about in-labour transfer from rural maternity care: a social judgment and signal detection analysis. BMC Med Inform Decis Mak. 2012;12:122. DOI: 10.1186/1472-6947-12-122 23114289 PMC3536665

[8] Christensen-Szalanski JJ Diehr PH Bushyhead JB Wood RW. Two studies of good clinical judgment. Med Decis Making. 1982;2(3):275. 6133204 10.1177/0272989X8200200303

[9] Burton CD McLernon DJ Lee AJ Murchie P. Distinguishing variation in referral accuracy from referral threshold: analysis of a national dataset of referrals for suspected cancer. BMJ Open. 2017;7(8):e016439. DOI: 10.1136/bmjopen-2017-016439 PMC5629656 28827254

[10] Swets JA Dawes RM Monahan J. Psychological science can improve diagnostic decisions. Psychol Sci Public Interest. 2000;1(1):1–26. 26151979 10.1111/1529-1006.001

[11] Reyna VF Edelson S Hayes B Garavito D. Supporting health and medical decision making: findings and insights from fuzzy-trace theory. Med Decis Making. 2022;42(6):741–54. DOI: 10.1177/0272989X221105473 PMC9283268 35735225

[12] Reyna VF. A new intuitionism: meaning, memory, and development in fuzzy-trace theory. Judgm Decis Mak. 2012;7(3):332–59. DOI: 10.1017/S1930297500002291 PMC4268540 25530822

[13] Klein EY Martinez EM May L Saheed M Reyna V Broniatowski DA . Categorical risk perception drives variability in antibiotic prescribing in the emergency department: a mixed methods observational study. J Gen Intern Med. 2017;32(10):1083–9. DOI: 10.1007/s11606-017-4099-6 PMC5602760 28634909

[14] Kostopoulou O Kavleen A Palfi B. Using cancer risk algorithms to improve risk estimates and referral decisions. Commun Med. 2022;2:2. DOI: 10.1038/s43856-021-00069-1 35603307 PMC9053195

[15] National Institute for Health and Care Excellence (NICE). Suspected Cancer: Recognition and Referral. Final scope. July 16, 2024. Available from: https://alpha.nice.org.uk/guidance/GID-HTE10050/documents/html-to-pdf-7.

[16] Barratt H Kirwan M. Clustered data - effects on sample size and approaches to analysis. 2009. Available from: http://www.healthknowledge.org.uk/public-health-textbook/research-methods/1a-epidemiology/clustered-data.

[17] Reyna VF Lloyd FJ. Physician decision making and cardiac risk: effects of knowledge, risk perception, risk tolerance, and fuzzy processing. J Exp Psychol Appl. 2006;12(3):175–9. 10.1037/1076-898X.12.3.179 16953744

[18] Grol R Whitfield M De Maeseneer J Mokkink H. Attitudes to risk taking in medical decision making among British, Dutch and Belgian general practitioners. Br J Gen Pract J R Coll Gen Pract. 1990;40(333):134–6. PMC1371238 2115347

[19] Dienes Z. Using Bayes to get the most out of non-significant results. Front Psychol. 2014;5:781. 25120503 10.3389/fpsyg.2014.00781 PMC4114196

[20] Rouder JN Speckman PL Sun D Morey RD Iverson G. Bayesian t tests for accepting and rejecting the null hypothesis. Psychon Bull Rev. 2009;16:225–37. 10.3758/PBR.16.2.225 19293088

[21] Dawes RM. Social dilemmas. Annu Rev Psychol. 1980;31(1):169–93. DOI: 10.1146/annurev.ps.31.020180.001125

[22] Bicchieri C Dimant E. Nudging with care: the risks and benefits of social information. Public Choice. 2022;191(3–4):443–64. DOI: 10.1007/s11127-019-00684-6

[23] Meeker D Linder JA Fox CR , et al. Effect of behavioral interventions on inappropriate antibiotic prescribing among primary care practices. JAMA. 2016;315(6):562. DOI: 10.1001/jama.2016.0275 26864410 PMC6689234

[24] Hallsworth M Chadborn T Sallis A , et al. Provision of social norm feedback to high prescribers of antibiotics in general practice: a pragmatic national randomised controlled trial. Lancet. 2016;387(10029):1743–52. DOI: 10.1016/S0140-6736(16)00215-4 PMC4842844 26898856

[25] Robinson PJ Wouter Botzen WJ. The impact of regret and worry on the threshold level of concern for flood insurance demand: evidence from Dutch homeowners. Judgm Decis Mak. 2018;13(2):237–45.

[26] Kostopoulou O. Measuring gist-based perceptions of medication benefit-to-harm ratios. BMJ Qual Saf. 2024;33(10):622–3. DOI: 10.1136/bmjqs-2024-017375 39084905

[27] Nurek M Kostopoulou O. How the UK public views the use of diagnostic decision aids by physicians: a vignette-based experiment. J Am Med Inform Assoc. 2023;30(5):888–98. DOI: 10.1093/jamia/ocad019 PMC10114121 36795074

