# Supplementary material for: Determinants of Physicians’ Referrals for Suspected Cancer Given a Risk-Prediction Algorithm: Linking Signal Detection and Fuzzy Trace Theory
Source: Med Decis Making. 2025 Oct 16;46(1):88–101. doi: 10.1177/0272989X251376024 (PMC12705883; doi:10.1177/0272989X251376024)
Supplement: sj-pdf-1-mdm-10.1177_0272989X251376024 – Supplemental material for Determinants of Physicians’ Referrals for Suspected Cancer Given a Risk-Prediction Algorithm: Linking Signal Detection and Fuzzy Trace Theory [file sj-pdf-1-mdm-10.1177_0272989X251376024.pdf]

Supplementary Materials

⇒ Supplement 1: The vignettes

PRACTICE VIGNETTES

NAME: Bryony Barnes (female)

Age: 56

BMI: 30

Smoking: Currently smokes 15 cigarettes/day

Alcohol intake: 21 units/week

Age of menopause: 51

Bryony Barnes comes to see you complaining of being more constipated in the last month. Over the last 2 months, she has also noted that she has lost about 4kg in weight and doesn't understand why. She has not been dieting and her lifestyle has not changed.

QCANCER RISK: 1.04%

Appropriate response: No referral – risk <3%

NAME: Henry Lipp (male)

Age: 75

BMI: 24.9

Smoking: Never smoked

Alcohol intake: 4 units/week

Mr Henry Lipp has come to see you concerned because he noticed some blood in his stools over the last four weeks. He has no other symptoms.

QCANCER RISK: 6.33%

Appropriate response: Referral – risk >3%

NAME: Dawn Jenkins (female)

Age: 70

BMI: 26.2

Smoking: Ex-smoker

Alcohol intake: 14 units/week

Dawn Jenkins is your next patient. She has a background of Type 2 Diabetes and no other medical problems. She has come to see you because in the last few weeks she has become increasingly aware of some abdominal pain. When you ask her about her bowels, she says she has seen some blood in her stools on and off and this has been the case for the last two weeks. She has no other symptoms. You ask her to have some blood tests done which reveal a microcytic anaemia (Hb 9.8) and a low ferritin.

QCANCER RISK: 39.58%

Appropriate response: Referral – risk >3%

MAIN STUDY VIGNETTES

NAME: Adam Harper (male)

Age: 57

BMI: 24.6

Smoking: Never smoked

Alcohol intake: 21 units/week

Adam Harper is new to the practice. He comes to see you because his bowels are 'acting up'. On further questioning, he says that in the last few weeks his motions tend to be loose and he is opening his bowels more frequently. He says that he has always been very regular, going once a day, 'like clockwork'. He denies any change in his diet. He has no other symptoms and examination findings are normal.

QCANCER RISK: 0.69%

Appropriate response: No referral – risk <3%

NAME: Matt Crayton (male)

Age: 75

BMI: 24.4

Smoking: Never smoked

Alcohol intake: 4 units/week

Matt Crayton comes in to see you with his wife. He is seeking your advice because he has lost some weight recently. His wife intervenes and says that she and his friends have noticed this over the last 3 months and told him to see the GP. Matt says that he has not changed his diet. He has no other symptoms and examination findings are normal.

QCANCER RISK: 1.08%

Appropriate response: No referral – risk <3%

NAME: Nina Durbridge (female)

Age: 54

BMI: 27.2

Smoking: Ex-smoker

Alcohol intake: 21 units/week

Age of menopause: 51

Nina Durbridge, your next patient, works in marketing. She saw another doctor in your surgery last week and had requested to have some routine blood tests. She was called to come in for the results and has made an appointment for today. The results show microcytic anaemia (Hb 10.8) with a low ferritin. Upon enquiring about any symptoms that she may have, she tells you that she has had abdominal pain for about a month, which she has not had before. She has no other symptoms and examination findings are normal.

QCANCER RISK: 2.09%

Appropriate response: No referral – risk <3%

NAME: Antonio DiMarco (male)

Age: 78

BMI: 24.2

Smoking: Currently smokes 12 cigarettes/day

Alcohol intake: Nil

Antonio DiMarco comes to see you because he's lost some weight recently without dieting. His wife remarked upon it. Antonio also mentions that he's been passing stool more frequently than usual, which worries him a little because his father died of gastrointestinal cancer. He has no other symptoms and examination findings are normal.

QCANCER RISK: 5.16%

Appropriate response: Referral – risk >3%

NAME: Debbie Lawrence (female)

Age: 58

BMI: 21.8

Smoking: Currently smokes 3 cigarettes/day

Alcohol intake: 3 units/week

Age of menopause: 52

Debbie Lawrence comes in accompanied by her husband. She complains of abdominal pain which she has had for more than a month. She says that she cannot understand what might be causing it and that it is not getting better. She has also noticed that she is passing stool more frequently than usual (2 or 3 times a day) in the last few weeks. You order a blood test, which comes back showing microcytic anaemia (Hb 10.3) with low ferritin. She has no other symptoms and examination findings are normal.

QCANCER RISK: 4.70%

Appropriate response: Referral – risk >3%

NAME: Doris Newman

Age: 75

BMI: 29.4

Smoking: non-smoker

Alcohol intake: nil

Doris Newman has come to see you concerned about some abdominal pain that she has experienced in the last few weeks. It seems to be there most of the time. She says that she loves her food but has lost her appetite lately. She likes to check her weight regularly and has also noticed that it has dropped from 55kg to 52kg in the last month. On further questioning, she reveals that her bowels are opening less regularly in the last few weeks, and she finds it more difficult to pass stool. She has no other symptoms and examination findings are normal.

QCANCER RISK: 8.78%

Appropriate response: Referral – risk >3%

NAME: Norman England (male)

Age: 70

BMI: 26.7

Smoking: Current smoker, 5 cigarettes/day

Alcohol intake: 14 units/week

Norman England has come to see you because he has noticed that his stools have become loose and he is opening his bowels more frequently over the last four weeks. He usually loves his wife's cooking but doesn't feel like eating anymore. He has lost about 4kg of weight in the last 2 months. He has also noticed that he is getting some abdominal pain. He tells you that his father had bowel cancer when he was of a similar age. He has no other symptoms and examination findings are normal.

QCANCER RISK: 22.82%

Appropriate response: Referral – risk >3%

NAME: Jane Tarley (female)

Age: 75

BMI: 24.9

Smoking: Currently smokes 20 cigarettes/day

Alcohol intake: Nil

Jane Tarley is your next patient. She has a background of COPD for which she takes inhalers. She has become aware of some abdominal pain in the last month. She tells you that she is off her food, and thinks that she has lost weight. You weigh her and note that she has lost about 3kg in the last 2 months. Jane also tells you that she has had some blood in her stool most days in the last 2 weeks. She has not had anything like this before. She tells you that her brother was recently diagnosed with bowel cancer. She has no other symptoms and examination findings are normal.

QCANCER RISK: 40.14%

Appropriate response: Referral – risk >3%

NAME: Olivia Fielding (female)

Age: 88

BMI: 23.1

Smoking: ex-smoker

Alcohol intake: nil

Olivia Fielding comes in to see you today. She is usually well and has no significant past medical history. She tells you that her family have commented that she appears to have lost quite a bit of weight over the past 6 months. She has noticed that her clothes feel looser. On further questioning, you discover that she has had abdominal pain for most days in the last 3 months. Her stools seem to be 'more runny' in the last few weeks. You organise some blood tests, which reveal microcytic anaemia (Hb 10.1) and low ferritin. She has no other symptoms and examination findings are normal.

QCANCER RISK: 20.76%

Appropriate response: Referral – risk >3%

Supplement 2: Bayesian analyses

For the non-significant statistical tests, we used the Bayes factor (BF) (Dienes, 2014; Rouder et al. 2009) to distinguish between data insensitivity and evidence for the null or the alternative hypothesis. We applied the Dienes and Mclatchie Bayes factor calculator (2018) adopted to the R environment. We report BFs in the following format: $BF_{H(0, SD)}$, where H indicates that we modelled the predictions of the alternative hypotheses with half-normal distributions. The values within the parentheses indicate the parameters of the half-normal priors: 0 for the mode and SD for the SD of the distribution (for more information on how we defined the SDs for each hypothesis, see below). While the BF is a continuous measure of evidence, we used it for hypothesis testing by applying the conventional threshold of 3 for substantial evidence for the alternative hypothesis, and the threshold of 1/3 for substantial evidence for the null hypothesis. We interpreted BFs between 3 and 1/3 to indicate data insensitivity (Jeffreys, 1961). To ascertain the robustness of our conclusions to the parameters of our models, we report Robustness Regions (RRs) for each BF, where we indicate the range of SDs for which we would have arrived to the same qualitative conclusion (Dienes, 2019).

The predictions of all alternative hypotheses (also known as prior distributions) were modelled with half-normal distributions with a mode of zero; hence, we only needed to identify the SD of these distributions for the various hypotheses. Notably, the SD of such a distribution represents the effect size one would expect if the alternative hypothesis were true (Dienes, 2019). We used two heuristics to identify the expected effect size for each hypothesis. If there was a relevant effect in the literature, we used the effect size found in a previous study. If there was no relevant effect in the literature, we used the "room-to-move heuristic", which recommends using the half of the maximum possible effect size as the expected effect size (Dienes, 2019). We used the largest relevant effect size with a significant test for the maximum possible effect size. Here, we list the applied heuristic for each hypothesis:

H1: all tests were statistically significant, so we did not run a Bayesian analysis
H2: we used the relevant effect size from Kostopoulou et al. (2022), OR = 1.26, as the expected effect size.
H3 and H6: perceived global patient benefits were the strongest predictor of referral likelihood with a coefficient of $b = 0.30$. We used this value as the maximum possible effect size for the non-significant predictors; hence the expected effect size was 0.15 for all non-significant predictors.
H4: the test was statistically significant, so we did not run a Bayesian analysis.
H5 and H7: Since the test of H4 was significant, we used the effect size of $b = 0.004$ as a potential maximum effect for H5 and H7. First, we halved the effect size and then converted it to the measurement scales used for H5 and H7 by multiplying it by 2 and 50, respectively. The expected effect sizes for H5 and H7 were $b = 0.004$ and $b = 0.10$ respectively.

Dienes, Z. (2019). How Do I Know What My Theory Predicts? *Advances in Methods and Practices in Psychological Science*, *2*(4), 364–377. https://doi.org/10.1177/2515245919876960

Dienes Z. Using Bayes to get the most out of non-significant results. *Front Psychol*. 2014;5:781.

Rouder JN, Speckman PL, Sun D, Morey RD, Iverson G. Bayesian t tests for accepting and rejecting the null hypothesis. *Psychon Bull Rev*. 2009;16:225-237.

Jeffreys H. *The Theory of Probability*. Oxford University Press; 1961.

Kostopoulou, O., Kavleen, A., & Palfi, B. (2022). Using cancer risk algorithms to improve risk estimates and referral decisions. *Communications Medicine*, *2*(2). https://doi.org/10.1038/s43856-021-00069-1

⇒ Supplement 3: Perceived harms and benefits as predictors of referral responses

Regression coefficients, 95% confidence intervals, and p values of multiple regression models predicting referral responses pre-algorithm and post-algorithm.

Stakeholder	Predictor	Pre-algorithm	Post-algorithm	Predictor x Timing (pre-post) interaction
**Patient**	Benefits	*b*=0.32 [0.23, 0.40] *p*<0.001	*b*=0.23 [0.14, 0.31] p<0.001	*b*=0.01 [-0.08, 0.10] p=0.752 $BF_{H(0, 0.15)}$ = 0.23
	Harms	*b*=-0.08 [-0.17, 0.006] *p*=0.067	*b*=-0.04 [-0.13, 0.05] *p*=0.351	*b*=0.02 [-0.07, 0.12] p=0.600 $BF_{H(0, 0.15)}$ = 0.47
**NHS/Society**	Benefits	*b*=0.23 [0.15, 0.32] *p*<0.001	*b*=0.17 [0.08, 0.26] *p*<0.001	*b*=-0.06 [-0.15, 0.03] p=0.163 $BF_{H(0, 0.15)}$ = 1.29
	Harms	*b*=-0.15 [-0.25, -0.06] *p*=0.002	*b*=-0.12 [-0.22, -0.03] *p*=0.012	*b*=-0.01 [-0.11, 0.10] p=0.928 $BF_{H(0, 0.15)}$ = 0.32
**GP/Practice**	Benefits	*b*=-0.0004 [-0.09, 0.09] *p*=0.993	*b*=-0.009 [-0.10, 0.08] *p*=0.845	*b*=0.04 [-0.04, 0.13] p=0.330 $BF_{H(0, 0.15)}$ = 0.15
	Harms	*b*=-0.04 [-0.14, 0.06] *p*=0.427	*b*=0.01 [-0.09, 0.11] *p*=0.809	*b*=-0.001 [-0.10, 0.10] p=0.991 $BF_{H(0, 0.15)}$ = 0.32

NB. The Bayesian analyses of the interactions revealed evidence for the null for the impact of the algorithm on patient benefits, GP benefits, GP harms, and NHS harms, while the rest of the analyses showed data insensitivity.

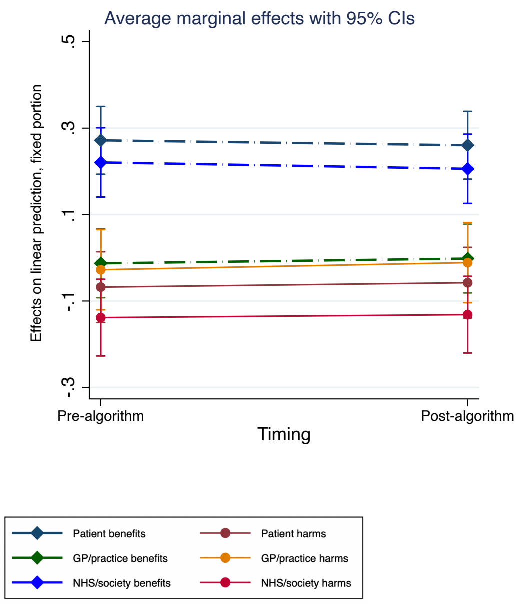

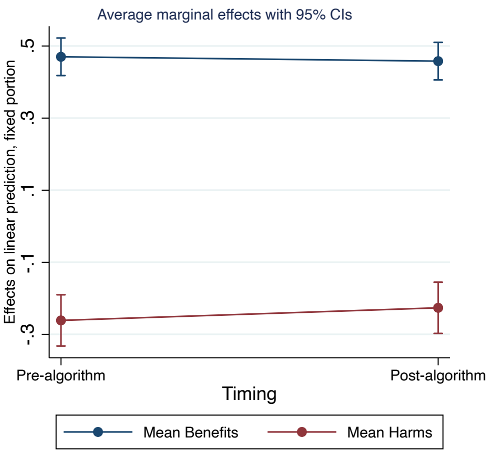

⇒ Supplement 4: GP comments

GP comments written at the end of the survey, which suggest that GPs interpreted the harm/benefit questions in different ways, and some had difficulty thinking about referrals in those terms.

"Grading at the end is difficult - is it a potential harm or benefit for a patient to be diagnosed with cancer?" (GP 177)

"I liked that you had a section on harms of overdiagnosis." (GP 22)

"Very interesting to think about the potential benefits to patient and society following referrals. The potential benefits are theoretically higher for the patient if their risk of cancer is low, and they are referred for those rarer diagnoses. Conversely the benefit to society for referral reaches a peak with higher risk cases." (pilot participant 2)

"I didn't really understand the second part of the survey where we had to say how negligible, low, medium and high-risk patients being a referred on a 2WW pathway would be harmful/beneficial to the NHS/practice." (GP 145)

"I found the benefit to GP/practice question difficult to answer. I wasn't sure what I was meant to be thinking about when answering this question." (pilot participant 1)

⇒ Supplement 5: Ordinal logistic regression analyses

Given that our main outcome variables, referral responses, risk judgements and perceived harms and benefits were measured on scales with limited width, where distances between the units are not necessarily equal, we repeated the regression analyses assuming that our outcome variables are ordinal, to assess the robustness of our conclusions. We ran cumulative link mixed models using the ordinal R package (Christensen, 2023), and in each regression model, we included the same random effects variables as we did in their linear counterparts. For the ordinal predictors of these models, we report the Odds ratios (ORs) of the linear trends of the polynomial contrasts. Note that these ORs cannot be interpreted in the usual way of linear regression (e.g., one unit change in the predictor leads to X change in the outcome measure). They indicate a trend (increasing or decreasing) across the ordered levels of the predictor. For example an OR>1 indicates the increase in the odds of being in a higher category of the outcome variable for each step along the linear trend of the predictor (e.g., from negligible to high in a steady increase).

Algorithm impact on risk and referral responses

In two separate regression models, we regressed referral responses and risk judgements on Timing (pre- vs. post-algorithm). In line with our main findings, we found that both the likelihood of referral and risk judgements reduced post-algorithm (referral *OR* 0.84 [0.73, 0.97] *p*=0.015, and risk judgement OR 0.53 [0.45, 0.61] *p*<0.001).

Perceived harms and benefits as a function of risk level

Regression coefficients of the linear trends reported as Odds Ratios (ORs), 95% Confidence Intervals (CIs), and p values of a multiple ordinal regression model predicting perceived harms and benefits as a function of risk level (risk judgement).

Outcome variable	OR	95% CI	p
**Benefits to patients**	4761	1620, 13995	<0.001
**Harms to patients**	0.013	0.008, 0.021	<0.001
**Benefits to NHS/Society**	978	453, 2107	<0.001
**Harms to NHS/Society**	0.009	0.005, 0.015	<0.001
**Benefits to GP/Practice**	966	438, 2134	<0.001
**Harms to GP/Practice**	0.033	0.020, 0.053	<0.001

Perceived harms and benefits as predictors of referral responses

Regression coefficients of the linear trends reported as Odds Ratios (ORs), 95% Confidence Intervals (CIs), and p values of a multiple ordinal regression model predicting referral responses.

Stakeholder	Predictor	OR	95% CI	p
**Patient**	Benefits	8.89	5.18, 15.24	<0.001
	Harms	0.47	0.28, 0.81	0.006
**NHS/Society**	Benefits	3.16	1.91, 5.25	<0.001
	Harms	0.21	0.11, 0.39	<0.001
**GP/Practice**	Benefits	1.39	0.81, 2.40	0.232
	Harms	1.65	0.68, 4.00	0.272
	Timing (post-algorithm)	1.11	0.96, 1.28	0.177

Perceived harms and benefits as predictors of referral responses pre- and post-algorithm

Regression coefficients of the linear trends reported as Odds Ratios (ORs), 95% Confidence Intervals (CIs), and p values of multiple ordinal regression models predicting referral responses pre-algorithm and post-algorithm.

Stakeholder	Predictor	Pre-algorithm	Post-algorithm
**Patient**	Benefits	*OR*=9.88 [4.59, 21.25] *p*<0.001	*OR*=6.52 [3.19, 13.35] p<0.001
	Harms	*OR*=0.46 [0.22, 0.99] *p*=0.048	*OR*=0.47 [0.23, 0.97] *p*=0.042
**NHS/Society**	Benefits	*OR*=4.06 [2.06, 7.98] *p*<0.001	*OR*=1.98 [1.00, 3.93] *p*= 0.049
	Harms	*OR*=0.21 [0.09, 0.54] *p*=0.001	*OR*=0.26 [0.11, 0.59] *p*=0.001
**GP/Practice**	Benefits	*OR*=1.12 [0.56, 2.25] *p*=0.746	*OR*=1.65 [0.81, 3.37] *p*=0.167
	Harms	*OR*=3.11 [0.78, 12.39] *p*=0.107	*OR*=1.27 [0.41, 3.93] *p*=0.672

Christensen, R. H. B. (2023). ordinal—regression models for ordinal data. *R package version*, 2023.*12-4.1*. https://cran.r-project.org/web/packages/ordinal/index.html
